# The Added Value of Genome-Wide Copy Numbers to Objectively Resolve Clonality of Multiple Tumors With Pulmonary Involvement and Ambiguous or Inconclusive Mutational Diagnosis

**DOI:** 10.1016/j.jtocrr.2025.100921

**Published:** 2025-10-23

**Authors:** Jurriaan Janssen, Bárbara Andrade Barbosa, Tim R. Mocking, Hendrik F. van Essen, Paul P. Eijk, Jacqueline Egthuijsen, Anabela Ferro, Jose-Pedro Parracha de Matos, Swip Draijer, Albrecht Stenzinger, Anke van den Berg, José Carlos Machado, Erik Thunnissen, Yongsoo Kim, Teodora Radonic, Bauke Ylstra

**Affiliations:** a Department of Pathology, Cancer Center Amsterdam, Amsterdam UMC, Vrije Universiteit Amsterdam, Amsterdam, The Netherlands; b Department of Hematology, Cancer Center Amsterdam, Amsterdam UMC, Vrije Universiteit Amsterdam, Amsterdam, The Netherlands; c Institute of Molecular Pathology and Immunology of the University of Porto (Ipatimup), Porto, Portugal; d Institute for Research and Innovation in Health (i3S), Porto, Portugal; e Institute of Pathology, University Hospital Heidelberg, Heidelberg, Germany; f Department of Pathology and Medical Biology, University of Groningen, University Medical Center Groningen, Groningen, The Netherlands; g Faculty of Medicine of the University of Porto, Porto, Portugal

**Keywords:** Clonal relationship, Multiple cancers, Mutations, Metastatic disease, Chromosomal copy number aberrations, Lung carcinoma

## Abstract

**Introduction:**

The incidence of patients presenting with multiple cancers (MCs) and pulmonary involvement is increasing. Although next-generation sequencing mutation panels can discern metastases (clonal) from separate primary cancers (nonclonal), it does not warrant a reliable diagnosis for all patients despite significant therapeutic implications. We evaluated the added value of genome-wide copy number aberrations (CNAs) for clonality diagnosis.

**Methods:**

Two cohorts were assembled: 41 clonal and 41 nonclonal pairs from the TRACERx cohort and 21 MC pairs that had sufficient DNA for whole-exome sequencing (WES) from 120 patients diagnosed using CNA analysis in our routine pathology practice between 2016 and 2022. Clonality was determined by comparing tumor pairs using (1) WES mutations as a definitive standard, (2) a conventional mutation panel with an adapted 2024 International Association for the Study of Lung Cancer algorithm, and (3) CNAs with log-likelihood ratio and Pearson correlation metrics.

**Results:**

All tumor pairs classified as definite “clonal” or “nonclonal” by mutation analysis (TRACERx: 35 of 82 [43%], MC cohort: 6 of 21 [29%]) were in concordance with WES and CNAs. Of the tumor pairs classified as “probable nonclonal” or “inconclusive” by mutation analysis (TRACERx: 47 of 82 [57%], MC cohort: 15 of 21 [71%]), most could be correctly reclassified by CNAs (TRACERx: 46 of 47 [98%], MC cohort: 15 of 16 [94%]). For each cohort, one tumor pair remained inconclusive. Furthermore, we present a CNA clonality workflow for implementation in molecular diagnostics.

**Conclusion:**

Genome-wide CNA analysis provides complementary information to resolve clonality of MCs with ambiguous mutational clonality status, enhancing clinical decision-making.

## Introduction

Multiple primary cancers, either synchronous or metachronous, can be as frequent as 20%, particularly for those patients with pulmonary involvement.^1^, ^2^, ^3^, ^4^, ^5^ Concordance of genetic mutations in multiple cancers (MCs) has become the mainstay to aid in diagnosis of their clonal relationship. In 2024, the International Association for the Study of Lung Cancer (IASLC) proposed a decision tree that divides MCs based on the mutations in the following four categories; intrapulmonary metastases (IPM, clonal), separate primary lung cancers (SPLCs, nonclonal), probable SPLCs, and inconclusive.^6^ The decision tree was, however, not designed for the common MC scenario where additional organs besides the lung are involved, including head and neck carcinomas^7^ or other carcinomas.^8^

**Figure 1:**
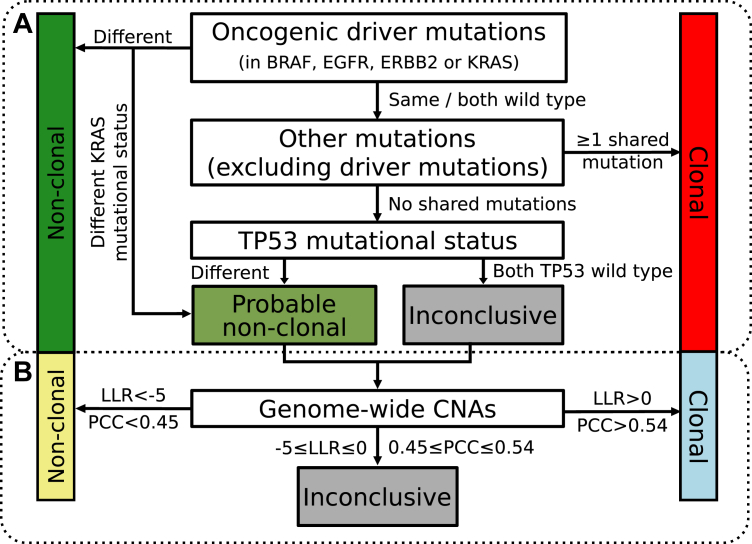
Clonality classification by molecular analysis. (*A*) Flowchart for objective assessment of clonality using mutations according to the adapted 2024 IASLC molecular classification algorithm^6^^,^^10^ (modified from Yang et al.^11^). (*B*) Flowchart extension for added value of genome-wide CNAs to resolve ambiguous mutational clonality calls, according to the two-metric CNA algorithm log-likelihood (LLR) and Pearson correlation coefficient (PCC) with predefined cutoffs to classify nonclonal (LLR < −5; PCC < 0.45), clonal (LLR > 0; PCC > 0.54), and inconclusive (−5 ≤ LLR ≤ 0; 0.45 ≤ PCC ≤ 0.54). CNA, copy number aberration; IASLC, International Association for the Study of Lung Cancer.

In daily routine practice, pathology institutes use the targeted next-generation sequencing (NGS) mutation panels that are in place for predictive biomarker testing of advanced-stage NSCLC to aid in the diagnosis of MCs with pulmonary involvement. The minimal set of genetic mutations covered by these NGS panels encompasses *BRAF*, *EGFR*, *ERBB2*, *KRAS*, and *MET*, laid down in the European national guidelines,^9^ and it requires *TP53* for the use of the IASLC decision tree.^6^ Recently, we proposed a minor adaptation to the IASLC molecular classification algorithm whereby discordant KRAS mutational status is classified as “probable nonclonal,” rather than definite “nonclonal,”^10^ given its observed subclonal occurrence (Fig. 1*A*).^12^ With a mutation panel of 27 genes, the adapted IASLC algorithm classifies approximately 50% of MC patients with an unambiguous diagnosis.^10^ With large mutation panels (exceeding 300 genes), the vast majority of cases can be classified as definite clonal or nonclonal irrespective of the algorithm.^11^^,^^13^ Yet no objective measures allow to fully resolve the “probable nonclonal” or “inconclusive” MCs, and therefore, orthogonal (molecular) information might be of additional value.

Detection of oncogenic fusions in ALK, ROS1, RET, and NRG1 is broadly implemented in routine diagnostics for predictive biomarker testing using immunohistochemistry, in situ hybridization, and DNA or RNA sequencing.^14^ Owing to their low prevalence, shared presence of oncogenic fusions between MCs could serve as a means to resolve clonality. However, this has not yet been systematically investigated and therefore not included in the IASLC molecular classification algorithm.^6^ Alternatively, both whole genome chromosomal breakpoints and genome-wide copy number aberrations (CNAs) are recommended by the IASLC as a means to resolve the clonality of MCs.^6^ Breakpoints of chromosomal rearrangements can be compared between MCs but are challenging to identify in routine diagnostics.^6^ However, genome-wide CNAs can be obtained in routine diagnostics by shallow whole-genome sequencing (sWGS) and the concordance of gains and losses applied to classify MCs. In short, one approach classifies MCs in three categories—“clonal,” “nonclonal,” and “inconclusive”—using the concordance of genome-wide CNAs (Fig. 1*B*).^15^^,^^16^ Nevertheless, it is currently not routinely implemented in most pathology institutes, and its place in the IASLC molecular classification algorithm is not specified.

In this report, we evaluate the added value of genome-wide CNAs to make a diagnosis for patients with probable or inconclusive mutational clonality and propose a laboratory alternative to generate genome-wide CNAs in parallel with NGS panels. We investigated two independent cohorts, 82 NSCLC pairs from the TRACERx cohort^12^ and 21 tumor pairs of patients diagnosed with MC at Amsterdam UMC between 2016 and 2022. To allow for an objective evaluation of these cohorts, we used (1) whole-exome sequencing (WES) as a gold standard reference,^17^ (2) selected a conventional mutation panel that had previously revealed high performance in combination with the adapted 2024 IASLC molecular classification algorithm,^10^ (Fig. 1*A*) and (3) performed genome-wide CNA analysis to evaluate its added value in addition to the selected conventional mutation panel (Fig. 1*B*).

## Materials and Methods

### Patient Cohorts

We used two patient cohorts, the TRACERx NSCLC cohort and an in-house diagnostics MC cohort. The TRACERx cohort data^12^ were made available through the European Genome-phenome Archive (EGA), accession number EGAS00001002247. The TRACERx data consist of WES data derived from biopsies of multiple regions in the same tumor from NSCLC. WES mutation data were obtained for 132 intratumor fresh frozen tumor biopsies. We constructed 41 clonal pairs by randomly sampling two biopsies of the same tumor per patient, representing IPM (clonal) pairs. We constructed 41 nonclonal pairs by randomly sampling two biopsies from different patients. Only tumors with the same histologic subtype, either lung adeno- (LUAD) or squamous cell carcinoma (LUSC), were paired (Supplementary Tables 1 and 2A). These artificial tumor pairs were created under the assumption that genetic differences between two primary tumors within one individual (nonclonal) are the same as between tumors of two individuals. Also, it was assumed that genetic similarities for multiple biopsies from the same tumor are not different compared with tumor and its metastasis (clonal).

**Figure 2:**
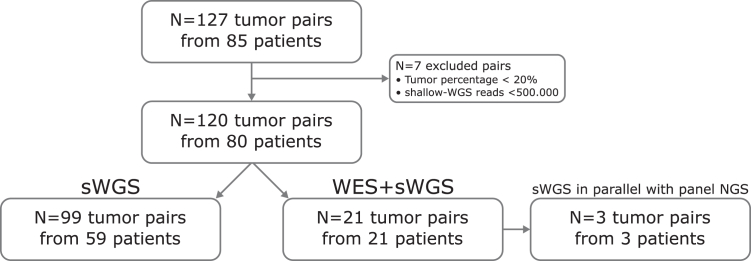
Flowchart of sample selection and exclusion for the in-house MC cohort, patients diagnosed using sWGS for CNA analysis in our routine pathology practice between 2016 and 2022. CNA, copy number aberration; MC, multiple cancer; NGS, next-generation sequencing; sWGS, shallow whole-genome sequencing; WES, whole-exome sequencing.

The in-house cohort consisted of all MC patients diagnosed between 2016 and 2022 for which clonality was unresolved with the clinicopathologic and NGS mutation information available at the time. These patients were all subjected to genome-wide CNA analysis, as recommended in the 2016 IASLC guidelines.^18^ We retrieved 127 tumor pairs from 85 patients, of which most were suspected to have pulmonary involvement (Fig. 2, Supplementary Table 3). Furthermore, 11 biopsies (6%) were excluded due to low tumor percentage (<20%) as determined by two experienced pathologists or insufficient sWGS read counts (<500,000 reads), leaving 80 patients and 120 tumor pairs for CNA clonality analysis (Fig. 2, Supplementary Table 3). Of these 120 tumor pairs (38% LUAD and 62% LUSC), 112 (93%) had pulmonary involvement and eight (7%) did not (Supplementary Table 3). Of these, 21 tumor pairs had sufficient DNA (>250 ng) available for additional WES mutation analysis (Fig. 1, Supplementary Table 3). Although statistical power is limited due to sample size, statistical analysis revealed that the 21 tumor pairs were not significantly different from the entire cohort in terms of clinicopathologic features (Supplementary Table 2B). After the WES analysis, three tumor pairs had sufficient DNA (>100 ng each sample) to perform additional sWGS on a newly developed laboratory workflow on a single flow-cell together with routine targeted panel NGS to demonstrate that comparable CNA results are obtained. Patient consent for the in-house MC cohort was waived by the Medical Ethics Review Committee of the Amsterdam University Medical Center (2022-0140). Diagnostic patient data could not be made public, as no explicit patient consent was given for data sharing.

### Mutations and Genome-Wide CNAs for the TRACERx Cohort

For the TRACERx cohort, WES mutation calls were used as published.^12^

Genome-wide CNAs were inferred by calculating the number of off-target sequence reads per 100 kbp bin from the WES using CopywriteR (version [v.] 2.20.0)^19^ following filtering and normalization steps as detailed in the Supplementary Methods. Genome-wide CNAs of the TRACERx were highly similar with the TCGA^20^^,^^21^ and concordant with previous reports,^22^, ^23^, ^24^ with gains of chromosomes 1q, 5p, 7, 8q, and 20 and losses of 6, 9, and 18 as the most common aberrations in LUAD and gains in chromosomes 3q, 5p, and 8q and losses in 3p, 5q, 8p, 9p, and 13 most common in LUSC (Supplementary Fig. 1*A–D*).

### Mutations and Genome-Wide CNAs for the In-House MC Cohort

For the in-house MC cohort, tumor macrodissection was performed on formalin-fixed, paraffin-embedded (FFPE) sections by either of two pathologists (T.R. and E.T.) aiming at a tumor cell percentage of more than 30% as described.^25^ WES was performed using a previously published standard pipeline^26^ for the 42 samples of the in-house MC cohort for which sufficient DNA (>250 ng) could be retrieved for both samples of the MC pair (Supplementary Methods). We extended the pipeline to filter out germline variants without a matched normal (Supplementary Fig. 2, Supplementary Methods). For genome-wide CNAs, DNA was isolated using the QIAamp DNA micro kit (Qiagen, Hilden, Germany), followed by whole genome library preparation and sequencing (Illumina San Diego, CA) as detailed in the Supplementary Methods with a target of 10 million reads per sample. A maximum of one MC pair (two samples) was run together with 94 non-invasive prenatal testing samples (total 96 samples) at Amsterdam UMC in our non-invasive prenatal testing diagnostic workflow that is performed biweekly.^27^ CNA analysis was performed using the QDNAseq R package version 1.24.0^28^ with a bin size of 100, 500, or 1000 kb depending on the obtained sequence depth, as detailed in the Supplementary Methods. Genome-wide CNAs of the in-house MC cohort were highly similar with the TCGA and TRACERx cohorts (Supplementary Fig. 1*A–F*).

### Clonality Class of Tumor Pairs With WES as a Gold Standard

Gold standard clonal relationship was determined for all tumor pairs using WES mutation data. Based on Liu et al.,^17^ we used a threshold of more than two mutations shared at DNA variant level to define true clonal versus true nonclonal tumor pairs.

### Clonality Class of Tumor Pairs With a Conventional Panel of Mutations

Tumor pairs were classified as “clonal,” “nonclonal,” “probable nonclonal,” or “inconclusive” based on a conventional mutation panel extracted from WES mutation calls to cover eight guideline oncogenes for predictive biomarker testing in NSCLC^9^ combined with 19 genes often mutated in NSCLC (Supplementary Table 4 and Supplementary Methods).

### Genome-Wide CNAs for the TCGA Reference Cohort

To correct clonality scores for frequently observed CNAs in NSCLC tumors, data of 200 LUAD and 200 LUSC from the TCGA cohort^20^^,^^21^ were downloaded as normalized copy number log_2_ ratios per segment through the public TCGA data portal (https://tcga-data.nci.nih.gov/tcga/tcgaHome2.jsp). These data were generated using genome-wide SNP arrays (Affymetrix Genome-Wide Human SNP Array 6.0). The CNA segments were also divided into 100 kbp bins, as used for the TRACERx and in-house diagnostics cohort, using QDNAseq,^28^ and CNA frequencies were calculated for LUAD and LUSC separately. CNA frequencies were calculated per chromosomal arm to use as a reference distribution for the log-likelihood ratio (LLR)^16^ calculations.

### Clonality Class of Tumor Pairs With CNA Profiles

Two separate and published metrics for tumor clonality using CNAs were combined for classification of clonality class (“clonal,” “nonclonal,” and “inconclusive”): Pearson correlation coefficient (PCC)^15^ and LLR.^16^ This two-metric clonality classifier was used in our routine clinical pathology practice at Amsterdam UMC, location VUmc, between 2016 and 2022.

LLR calculations were performed as described in the *Clonality* R package (v.1.38.0)^16^ using the segmentation values. The LLR is a measure that accounts for the frequency of CNAs in a reference cohort. The frequencies of CNAs per chromosomal arm were separately calculated for LUAD and LUSC in the TCGA to serve as a reference for adeno- and squamous cell carcinoma LLR calculations, respectively. PCC calculations were performed between all copy number values of both samples of each tumor pair for the purpose of determining tumor clonality, as described.^15^ We applied previously established thresholds for the PCC^15^ and LLR^16^ metrics: for “clonal” PCC more than 0.54 and LLR more than 0, for “nonclonal” PCC less than 0.45 and LLR less than −5, and an “inconclusive” gray area falling between these thresholds (Fig. 1*B*). These thresholds are those used in routine diagnostic practice at Amsterdam UMC.

### Implementation of Genome-Wide CNAs in Parallel With Routine Targeted Panel NGS in Molecular Pathology Diagnostics

We developed a workflow for sWGS in parallel with routine targeted panel NGS using the IonTorrent workflow, further detailed in the Supplementary Methods. With this workflow, each WGS sample on the Ion 540 chip (Thermo Fisher, Porto Salvo, Lisbon) yields an estimated 2 to 3 million single-end reads of 200 bp.

## Results

### WES Gold-Standard Clonality Class Compared With Mutational Panel Clonality Class in the TRACERx Cohort

Gold-standard clonality was verified for 41 clonal and 41 nonclonal tumor pairs in the TRACERx cohort with WES. In the TRACERx, the 41 clonal biopsy pairs had a median of 435 shared mutations (range: 100–2095) and the 41 nonclonal biopsies had a median of zero shared mutations (range: 0–2). After extracting the conventional mutation panel calls from the WES data, true clonal pairs had a median of one shared mutation (range: 0–5) and true nonclonal pairs had a median of zero shared mutations (range: 0–1).

**Figure 3:**
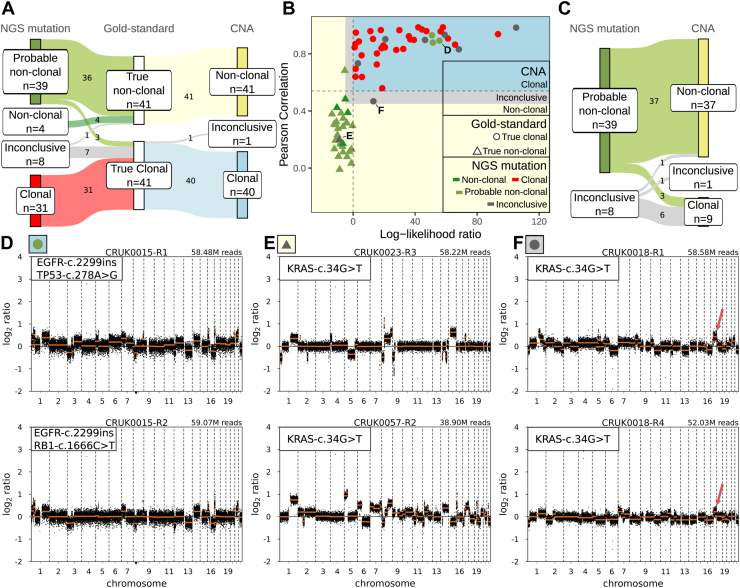
Performance of CNA- and conventional mutation panel-based clonality classification in the TRACERx gold-standard cohort. (*A*) Sankey diagram of clonality classification of tumor pairs by left, “NGS mutation,” conventional mutation panel alone, middle, “gold standard” by WES, and right, “CNA” CNAs alone. (*B*) Clonal and nonclonal tumor pair comparisons plotted by LLR, x-axis and PCC, y-axis. Dots indicate gold-standard clonal pairs by WES, and triangles indicate gold-standard nonclonal (interpatient) pairs by WES. The blue area (PCC > 0.54 and LLR > 0), yellow area (PCC < 0.45 and LLR < −5), and gray area (0.45 < PCC < 0.54 and 0 < LLR < −5) indicate, respectively, clonal, nonclonal, and inconclusive classification by the two-metric CNA classifier. Each pair is colored by the clonality determined by the conventional mutation panel (red: clonal, gray: inconclusive, green: nonclonal, olive green: probable nonclonal). (*C*) Sankey diagram of CNA class of 47 sample pairs with “inconclusive” or “probable nonclonal” classification by the conventional mutation panel. Examples of CNA profile pairs with discordance between the NGS and gold-standard clonality; (*D*) true clonal pair classified as “probable nonclonal” by the conventional mutation panel, (*E*) true clonal pair classified as inconclusive by the conventional mutation panel, and (*F*) true nonclonal pair classified as inconclusive by the conventional mutation panel. Copy number gains present in both tumors are denoted by red arrows. Black dots indicate log_2_ read counts (y-axis) used for the PCC calculation, and orange lines indicate segmentation values used for the LLR calculation. Total binned read counts for each profile are indicated in the top right. All exact mutations with amino acid position for each tumor pair detected in the conventional mutation panel are indicated in the top left. CNA-, NGS mutation- and gold-standard clonality for each pair is indicated in the top left according to the colors and shape legend in panel (*B*). LLR and PCC statistics corresponding to example pairs in panels (*D*) to (*F*) are indicated in panel (*B*). CNA, copy number aberration; LLR, log-likelihood ratio; NGS, next-generation sequencing; PCC, Pearson correlation coefficient; WES, whole-exome sequencing.

Subsequently, the adapted 2024 IASLC molecular classification algorithm (Fig. 1*A*) was used to classify “clonal,” “nonclonal,” “probable nonclonal,” and “inconclusive” using the conventional mutation panel calls. Of the 41 true nonclonal pairs, 36 were classified as “probable nonclonal” (88%), four as “nonclonal” (10%), and one as “inconclusive” (2%) (Fig. 3*A*, Supplementary Table 1). Of the 41 true clonal pairs, 31 were classified as “clonal” (76%), three as “probable nonclonal” (7%), and seven as “inconclusive” (17%). In total, 47 of 82 (57%) were classified as ambiguous (Fig. 3*A*). No association was observed between an ambiguous clonality and histologic subtype (*p* = 1.00, Fisher's exact test, Supplementary Table 5*A*). Of the three true clonal pairs classified as “probable nonclonal,” two were due to a different *KRAS* mutation status and one due to a different *TP53* mutation status (Supplementary Table 1).

### WES Gold-Standard Clonality Class Compared With CNA Clonality Class in the TRACERx Cohort

True clonal pairs (N = 41) had high genome-wide CNA concordance with a median PCC of 0.89 (range: 0.47–0.99) and a median LLR of 27.6 (range 0.77–05). True nonclonal pairs (N = 41) had low genome-wide CNA concordance with a median PCC of 0.28 (range: −0.01 to 0.68) and a median LLR of −6.84 (−13.6 to −0.387) (Fig. 3*B*).

Of the 41 true nonclonal pairs, all were classified as “nonclonal” (100%) with CNAs. Of the 41 true clonal pairs, 40 were classified as “clonal” (98%) and one as “inconclusive” (2%) (Fig. 3*A* and *B*, Supplementary Table 1). In total, 81 of 82 tumor pairs (99%) were classified as definite “clonal” or “nonclonal” and one of 82 (1%) as “inconclusive” with CNAs.

### The Added Value of CNA Analysis for Tumor Pairs With Inconclusive Clonality With the Conventional Mutation Panel in the TRACERx Cohort

All tumor pairs classified as definite “clonal” or “nonclonal” with the conventional mutation panel were classified to the same class with CNAs, in accordance with the gold standard (Fig. 3*B*).

The added value of genome-wide CNA analysis stems from the 47 tumor pairs classified as “inconclusive” or “probable nonclonal” with the conventional mutation panel (Fig. 3*C*). Of these, 46 pairs (98%) were classified as either “clonal” or “nonclonal” with CNA analysis, in accordance with the gold standard (Fig. 3*D* and *E*, Supplementary Fig. 3). The tumor pair classified as “inconclusive” by both the conventional mutation panel and CNAs was a true clonal pair with 388 shared somatic mutations. In both tumors, the same KRAS mutation and CNAs on chromosomes 1p, 10q, 16q, 17p, and 22 were observed, in addition to a multitude of discrepant CNAs (Fig. 3*F*).

### WES Gold-Standard Clonality Class Compared With Mutational Panel Clonality Class in the In-House MC Cohort

WES gold-standard clonality was determined for 21 tumor pairs of the in-house MC cohort. In nine tumor pairs, no shared somatic mutations were detected, which were classified as true nonclonal pairs (Supplementary Table 6). In the other 12 pairs, a median of 43 shared somatic mutations (range: 7–1326) was detected, which were classified as true clonal pairs (Supplementary Table 6). After extracting the conventional mutation panel calls from the WES data, true clonal pairs had a median of one shared mutation (range: 0–2) and true nonclonal pairs had a median of 0 shared mutation (range: 0–1) (Supplementary Table 6).

**Figure 4:**
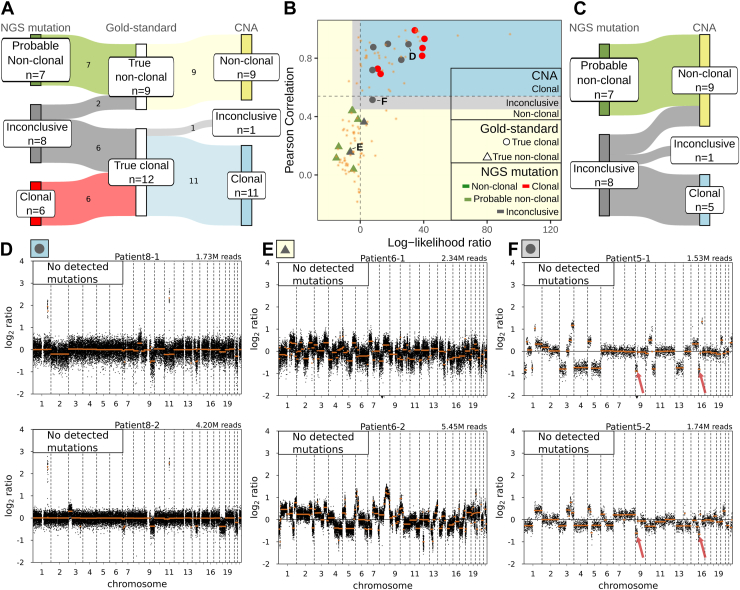
Performance of CNA- and mutation panel-based clonality classification in the diagnostic in-house MC cohort. (*A*) Sankey diagram of clonality classification by NGS mutations and CNA by sWGS compared with the gold-standard clonality. (*B*) Clonal and nonclonal comparisons plotted by LLR, x-axis and PCC, y-axis. Dots indicate gold standard clonal pairs by WES, triangles indicate gold-standard nonclonal (interpatient) pairs by WES, and small orange asterisks indicate pairs for which gold-standard WES clonality could not be performed due to insufficient DNA (flowchart in Fig. 2). The blue area (PCC > 0.54 and LLR > 0), yellow area (PCC < 0.45 and LLR < −5), and gray area (0.45 < PCC < 0.54 and 0 < LLR < −5) indicate, respectively clonal, nonclonal, and inconclusive classification by the two-metric CNA classifier. Each pair is colored by the clonality determined by the conventional NGS mutation panel (red: clonal, gray: inconclusive, green: nonclonal, olive green: probable nonclonal). (*C*) Sankey diagram of CNA class of 15 sample pairs with “inconclusive” or “probable nonclonal” classification by the conventional mutation panel. Examples of CNA profile pairs with discordance between the NGS mutation and gold-standard clonality; (*D*) true clonal pair classified as inconclusive by the conventional mutation panel, (*E*) true nonclonal pair classified as inconclusive by the conventional mutation panel, and (*F*) true clonal pair classified as inconclusive by both the conventional mutation panel and CNAs. Focal deletions present in both tumors are denoted by red arrows. Black dots indicate log_2_ read counts (y-axis) used for the PCC calculation, and orange lines indicate segmentation values used for the LLR calculation. Total binned read counts for each profile are indicated in the top right. The mutation calls overlapping the conventional mutation panel regions are indicated for each tumor in the top left. CNA-, NGS mutation- and gold-standard WES clonality for each pair is indicated in the top left according to the colors and shape legend in panel (*B*). LLR and PCC statistics corresponding to example tumor pairs in panels (*D*) to (*F*) are indicated in panel (*B*). CNA, copy number aberration; LLR, log-likelihood ratio; MC, multiple cancer; NGS, next-generation sequencing; PCC, Pearson correlation coefficient; sWGS, shallow whole-genome sequencing; WES, whole-exome sequencing.

Subsequently, the adapted 2024 IASLC molecular classification algorithm was used to classify tumor pairs using the conventional mutation panel calls (Fig. 1*A*). Of the nine true nonclonal pairs, seven were classified as “probable nonclonal” (78%) and two as “inconclusive” (22%) (Fig. 4*A*, Supplementary Table 3). Of the 12 true clonal pairs, six were classified as “clonal” (50%) and six as “inconclusive” (50%). In total, 15 of 21 (71%) were classified as ambiguous (Fig. 4*A*). No association was observed between ambiguous clonality and histologic subtype (*p* = 0.15, Fisher's exact test) or location (intrapulmonary versus extrapulmonary, *p* = 0.53, Supplementary Table 5*B* and *C*). Ambiguous clonality was significantly more frequently observed in metachronous versus synchronous MCs (*p* = 0.02, Supplementary Table 5*D*).

### WES Gold-Standard Clonality Class Compared With CNA Clonality Class in the In-House MC Cohort

CNA clonality class was determined with sWGS in routine diagnostics on weekly basis between 2016 and 2022. During this period, CNAs were used to classify clonality of the 120 diagnostic pairs, resulting in 49 “clonal” (41%), 70 “nonclonal” (58%), and four “inconclusive” (1%) tumor pairs (Fig. 4*B*, Supplementary Table 3). The comparison between the CNA clonality and true clonality was made only for the 21 tumor pairs for which gold-standard clonality was determined with WES.

Of the nine true nonclonal pairs, all were classified as “nonclonal” (100%) with CNAs. Of the 12 true clonal pairs, 11 were classified as “clonal” (92%) and one as “inconclusive” (8%) (Fig. 4*A*). In total, 20 of 21 tumor pairs (95%) were classified as definite “clonal” or “nonclonal” and one of 21 (5%) remained “inconclusive” with CNAs.

### The Added Value of CNA Analysis for Tumor Pairs With Inconclusive Clonality With the Conventional Mutation Panel in the In-House MC Cohort

Of 21 tumor pairs, six (29%) were classified as definite “clonal” or “nonclonal” with the conventional mutation panel calls classified to the same class with CNAs, in accordance with the gold standard (Fig. 4*B*). The added value of CNA analysis is demonstrated for the 15 pairs classified as “inconclusive” or “probable nonclonal” with the conventional mutation panel (Fig. 4*C*). Of these, 14 pairs (98%) were reclassified as either “clonal” or “nonclonal” with CNAs, in accordance with the gold standard (Fig. 4*C*–*E*, Supplementary Fig. 4). The clonal tumor pair classified as “inconclusive” by both the conventional mutation panel and CNAs was a synchronous, intrapulmonary pair (Fig. 4*F*) with 29 shared somatic mutations (Supplementary Table 6). Both tumors had CNAs on chromosomes 3p, 4, and 13 and shared focal deletions on 9p and 16p, in addition to a multitude of discrepant CNAs (Fig. 4*F*).

### Performance of sWGS for Genome-Wide CNAs in Parallel With Targeted Panel NGS in Routine Molecular Pathology Diagnostics

**Figure 5:**
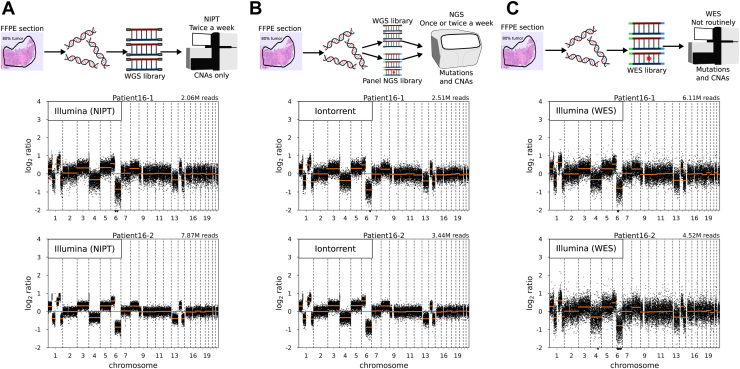
CNA profiles of a tumor pair using different sequencing platforms. Schematic laboratory workflow and CNA profiles of a diagnostic sample pair (*A*) by sWGS using the Illumina platform in parallel with NIPT, (*B*) by sWGS using the IonTorrent platform in parallel with NGS mutation panels, and (*C*) by WES off-target reads using the Illumina platform. Schemes in the top panels indicate from left to right the following: demarcated FFPE section example with estimated tumor cell percentage, DNA isolated from FFPE, fragmented and barcoded DNA and sequencing on a single flow cell. WGS and NGS libraries are created independently for panel (*B*) with separate barcodes (barcodes green and blue for panel NGS and WES; gray for WGS; the red star in the NGS/WES libraries symbolizes gene-specific mutation). For the two lower panels copy number profiles of tumor pairs: Black dots log_2_ read counts (y-axis) and orange lines segmentation values. Total binned read counts for each profile are indicated in the top right. This particular tumor pair was classified as clonal by WES, the conventional mutation panel, and CNAs. CNA profiles of the other two pairs analyzed are available in Supplementary Figure 5. CNA, copy number aberration; FFPE, formalin-fixed, paraffin-embedded; NIPT, non-invasive prenatal testing; NGS, next-generation sequencing; PCC, Pearson correlation coefficient; sWGS, shallow whole-genome sequencing; WES, whole-exome sequencing.

Although we have demonstrated the added value of CNAs, we acknowledge that sWGS analysis is not widely implemented in routine molecular pathology diagnostics. Therefore, we evaluated a workflow of sWGS together with routine targeted panel NGS on one sequencing chip using the IonTorrent platform for three in-house MC tumor pairs (Fig. 2). This yielded a sWGS read count of 2.5 to 3.7 million per sample (Supplementary Table 3) and near-identical CNA profiles (Fig. 5, Supplementary Fig. 5). Results of the other routine targeted panel NGS samples were not affected (diagnostics data not revealed).

## Discussion

Genome-wide CNA analysis can correctly reclassify the clonality of the vast majority of tumor pairs that were classified as “probable nonclonal” or “inconclusive” by the adapted 2024 IASLC algorithm with a conventional mutation panel.^6^^,^^10^ For each cohort, only one sample pair remained inconclusive by both mutation and CNA analysis, which could only be objectively determined using WES, and were both gold-standard clonal. Visual inspection of the CNA profiles revealed large discrepancies, but also distinct CNAs indicative of a clonal relationship were observed in both tumors of the pairs.

The fraction of tumor pairs classified as inconclusive was much higher for the in-house MC cohort (eight of 21 pairs, 40%) compared with the TRACERx cohort (eight of 82 pairs, 10%). This disparity likely arises because the TRACERx cohort is not representative of those found in routine diagnostic practice but is artificially constructed by taking intratumoral biopsy samples as clonal pairs and interpatient tumor samples as nonclonal pairs. The in-house MC cohort was therefore included in this study as it is more representative of those found during routine diagnostic practice, including extrapulmonary and metachronous samples, although biases cannot be excluded. Only sample pairs were submitted for CNA analysis if clonality classification could not be readily achieved by clinical mutations and/or histopathologic parameters at the time of diagnosis. The number of clonal sample pairs was 12 of 21 (57%) for the in-house MC cohort, of which five could only be resolved with CNA and one remained inconclusive, whereas the number of truly nonclonal tumor pairs of the house MC cohort was nine of 21 (43%), of which two could only be resolved by CNA. Taken together, the CNA test has clear added value to resolve inconclusive cases in both cohorts.

Practically, genome-wide CNA clonality analysis may be challenging to implement in diagnostics in a time- and material-efficient manner. However, we demonstrate that sWGS can be performed in a clinically timely manner by combining it with NGS panel analysis in routine diagnostic pathology laboratories.^28^ Thereby, sWGS can be performed with just 5 ng of DNA isolated from FFPE material.^29^ Conceivably, NGS panels capable of detecting both mutations and CNAs could serve as an alternative. However, such NGS panels are still not suitable for clonality classification because they lack genome-wide coverage (e.g., TSO500 only for amplifications^30^). To allow genome-wide detection of gains and losses in addition to amplifications, commercial CNA backbone spike-in panels could be used.

Large panels and, ultimately, WES are becoming increasingly accessible in daily pathology workflows, and it is plausible that they may serve as a gold standard for clonality assessment in the future. However, WES requires high DNA quality and quantity, including sequencing of normal tissue, which can lead to dropout rates of up to 25% in histology samples.^31^ For clonality comparison, mutations need to be strictly distinguished from germline variants, for which current WES processing tools also require a matched normal, thus germline, references samples. Such matched normal samples were not available for the retrospective in-house MC cohort either, which we addressed by selecting only the high confidence somatic mutations. This approach could also be implemented in routine MC WES analysis, if no matched normal is available. In lung pathology, predictive testing is particularly constrained by small biopsy sizes and a high proportion of cytology specimens,^32^ further limiting the clinical utility of WES for clonality assessment. Ultimately, any molecular diagnostic workflow is fundamentally constrained by the availability of adequate material, specifically tumor samples with a cellular content exceeding 10%^33^ and sufficient DNA input.

Independently of the laboratory method used, and to obtain high-quality CNA profiles, it is crucial that in the data analysis the chromosomal regions that display germline alterations or technical artifacts are filtered.^28^ Otherwise, germline alterations or technical artifacts appear in both tumors of a patient, whether clonal or nonclonal, and will thus hamper the distinction between the two.

In summary, genome-wide CNA analysis can provide crucial additional information to resolve the clonality status of tumors in patients with MCs, where ambiguity remains after standard diagnostic procedures such as NGS mutation panel analysis. This enhanced ability to distinguish between clonal tumors (metastases) and nonclonal tumors (separate primary cancers) can be a crucial addition to the repertoire of diagnostic tests for accurate disease staging and treatment strategies in patients with lung cancer with multiple lesions.

## CRediT Authorship Contribution Statement

**Jurriaan Janssen:** Methodology**,** Software, Validation, Formal analysis, Investigation, Data curation, Writing - original draft, Writing - review & editing, Visualization.

**Bárbara Andrade Barbosa:** Methodology, Software, Validation, Formal analysis, Investigation, Writing - original draft, Writing - review & editing, Visualization.

**Tim R. Mocking:** Methodology, Software, Validation, Formal analysis, Investigation, Writing - review & editing.

**Hendrik F. van Essen:** Validation, Investigation.

**Paul P. Eijk:** Validation, Investigation. Jacqueline Egthuijsen: Validation, Investigation.

**Jacqueline Egthuijsen:** Validation, Investigation.

**Anabela Ferro:** Writing - review & editing, Validation, Investigation.

**Jose-Pedro Parracha de Matos:** Methodology, Software, Validation, Formal analysis.

**Swip Draijer:** Writing - review & editing.

**Albrecht Stenzinger:** Writing - review & editing.

**Anke van den Berg:** Writing - review & editing.

**José Carlos Machado:** Resources, Writing - review & editing.

**Erik Thunnissen:** Methodology, Writing - review & editing.

**Yongsoo Kim:** Methodology, Investigation, Data curation, Writing - review & editing, Visualization, Supervision.

**Teodora Radonic:** Conceptualization, Formal analysis, Investigation, Resources, Writing - original draft, Writing - review & editing, Visualization, Supervision, Funding acquisition.

**Bauke Ylstra:** Conceptualization, Methodology, Formal analysis, Investigation, Writing - original draft, Writing - review & editing, Visualization, Supervision, Project administration, Funding acquisition.

## Disclosure

Dr. Stenzinger reports serving on the advisory board/speaker’s bureau of Agilent, Aignostics, Amgen, Astellas, AstraZeneca, Bayer, Bristol Myers Squibb, Eli Lilly, Illumina, Incyte, Janssen, Merck Sharp & Dohme, Novartis, Pfizer, Qlucore, QuiP, Roche, Sanofi, Seagen, Servier, Takeda, and Thermo Fisher. The remaining authors declare no conflict of interest.

## Supplementary Data

Supplemental_Material

SupplementaryTable 1

SupplementaryTable 2

SupplementaryTable 3

SupplementaryTable 4

SupplementaryTable 5

SupplementaryTable 6
