# Supplementary material for: The Added Value of Genome-Wide Copy Numbers to Objectively Resolve Clonality of Multiple Tumors With Pulmonary Involvement and Ambiguous or Inconclusive Mutational Diagnosis
Source: JTO Clin Res Rep. 2025 Oct 23;6(12):100921. doi: 10.1016/j.jtocrr.2025.100921 (PMC12681896; doi:10.1016/j.jtocrr.2025.100921)
Supplement: Supplemental_Material [file mmc1.docx]

## **Supplementary Figures**


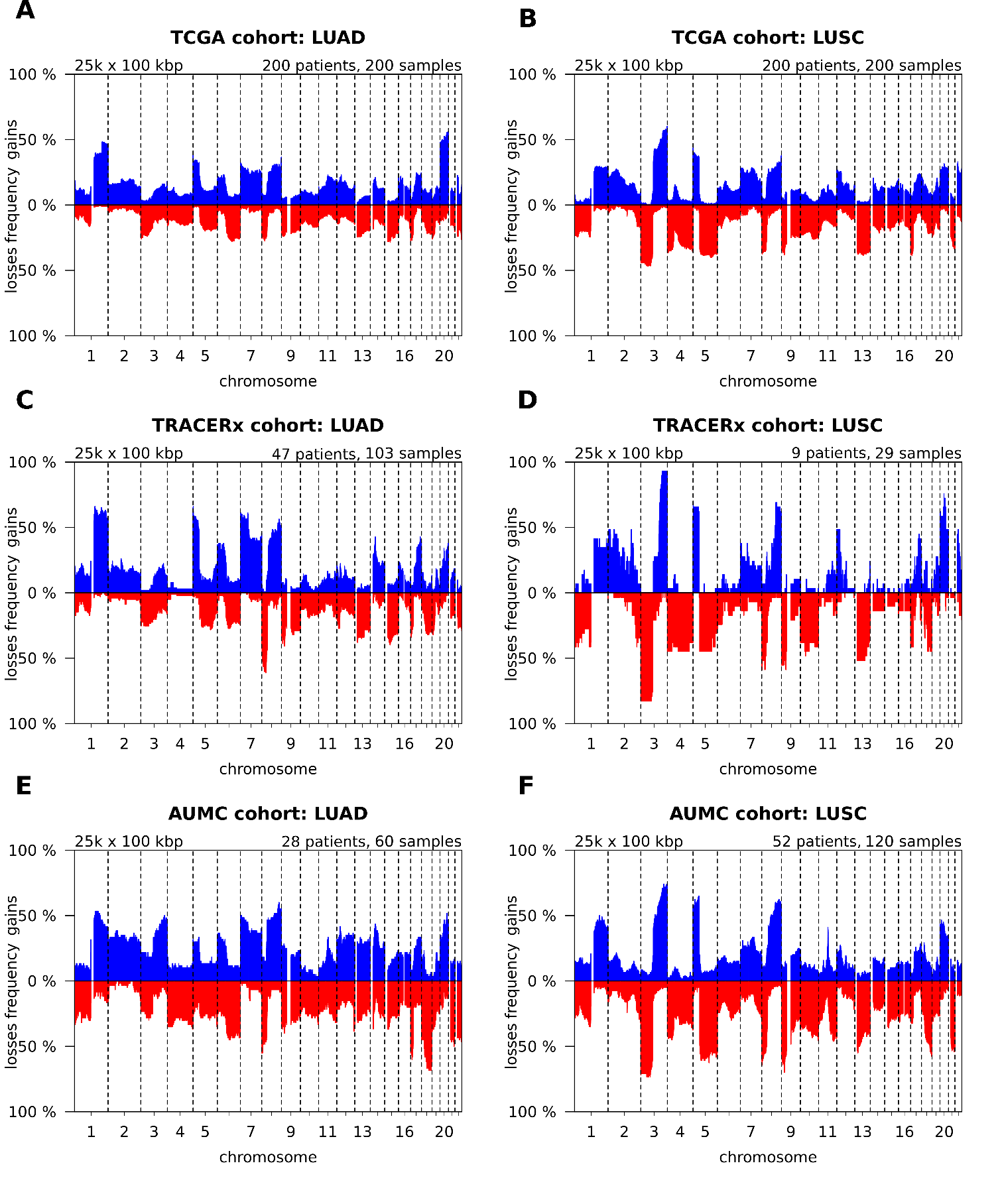


**Supplementary Figure 1. Copy number aberration (CNA) frequencies of LUSC and LUAD samples in study cohorts.** CNA frequencies in TCGA (A,B), TRACERx (C,D) and AUMC (E,F) cohorts for LUAD (A,C,E) and LUSC samples (B, D, F). CNAs were called using CGHcall with a cellularity of 0.75 for the TRACERx cohort and 0.25 for the other cohorts. Frequency of gains (red) and losses (blue) are shown on the y-axis, sorted in chromosomal order and by chromosomal position on the x-axis.

**
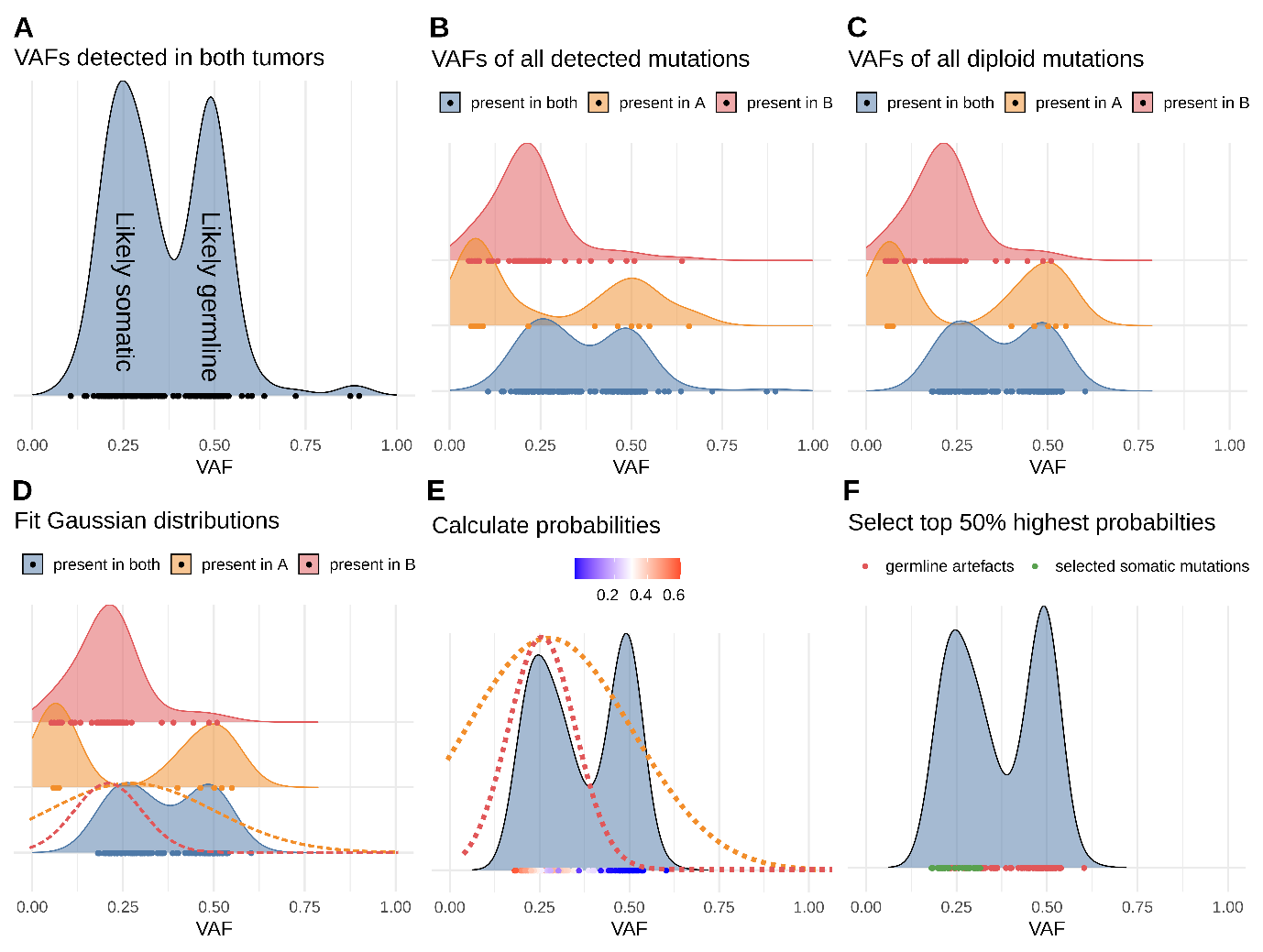
**

**Supplementary Figure 2. Filtering of germline variants without a matched-normal for the in-house MC cohort.** Figures depict the filtering procedure for the in-house MC cohort taking Patient 8 as an example. Density plot of the variant allele frequencies (VAFs) in detected overlapping mutations, which represent both somatic and germline variants (A). Density plot of VAFs in detected overlapping and non-overlapping mutations (B). Density plot of VAFs in detected overlapping and non-overlapping mutations in copy-neutral, diploid regions (C). Fitting of Gaussian distributions using the averages and standard deviations of non-overlapping mutations (D). Calculation of the probability using the Gaussian distribution as a probability density function as a measure for somatic confidence (E). Selection of somatic mutations using the top 50% highest somatic probabilities (F). The y-axes display the observed and modelled cumulative probability.


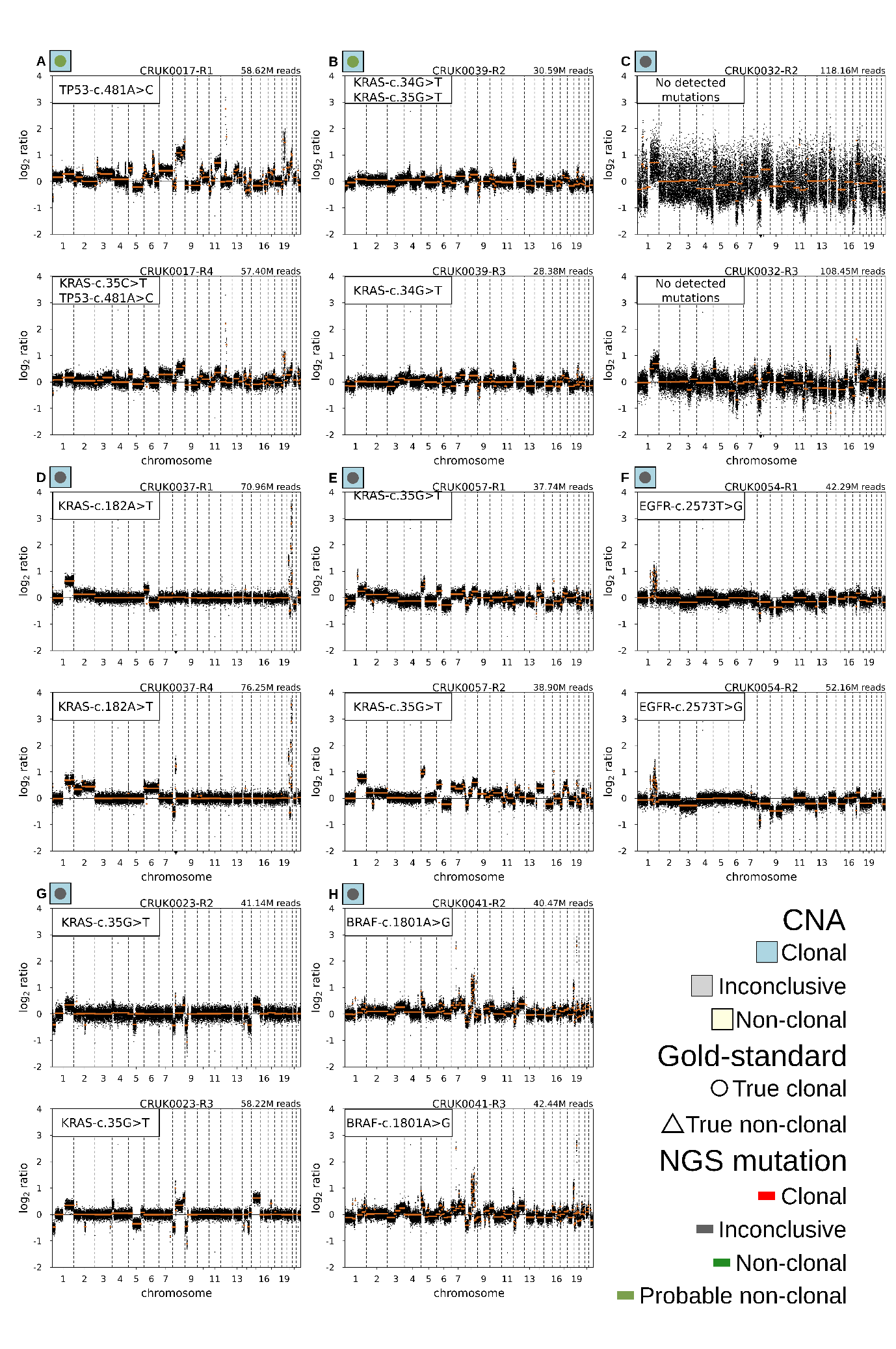


**Supplementary Figure 3. CNA profiles of TRACERx tumor pairs with inconclusive NGS mutation clonality or discordance with gold-standard clonality.** CNA profiles of TRACERx samples (A-H). CNA-, NGS mutation- and gold-standard clonality for each pair is indicated in the top left according to the color/shape legend in the bottom right. Black dots indicate log_2_ read counts (y-axis) and orange lines indicate segmentation values. Total binned read counts for each profile are indicated in the top right. The NGS mutation panel calls are indicated for each tumor in the top left.

**
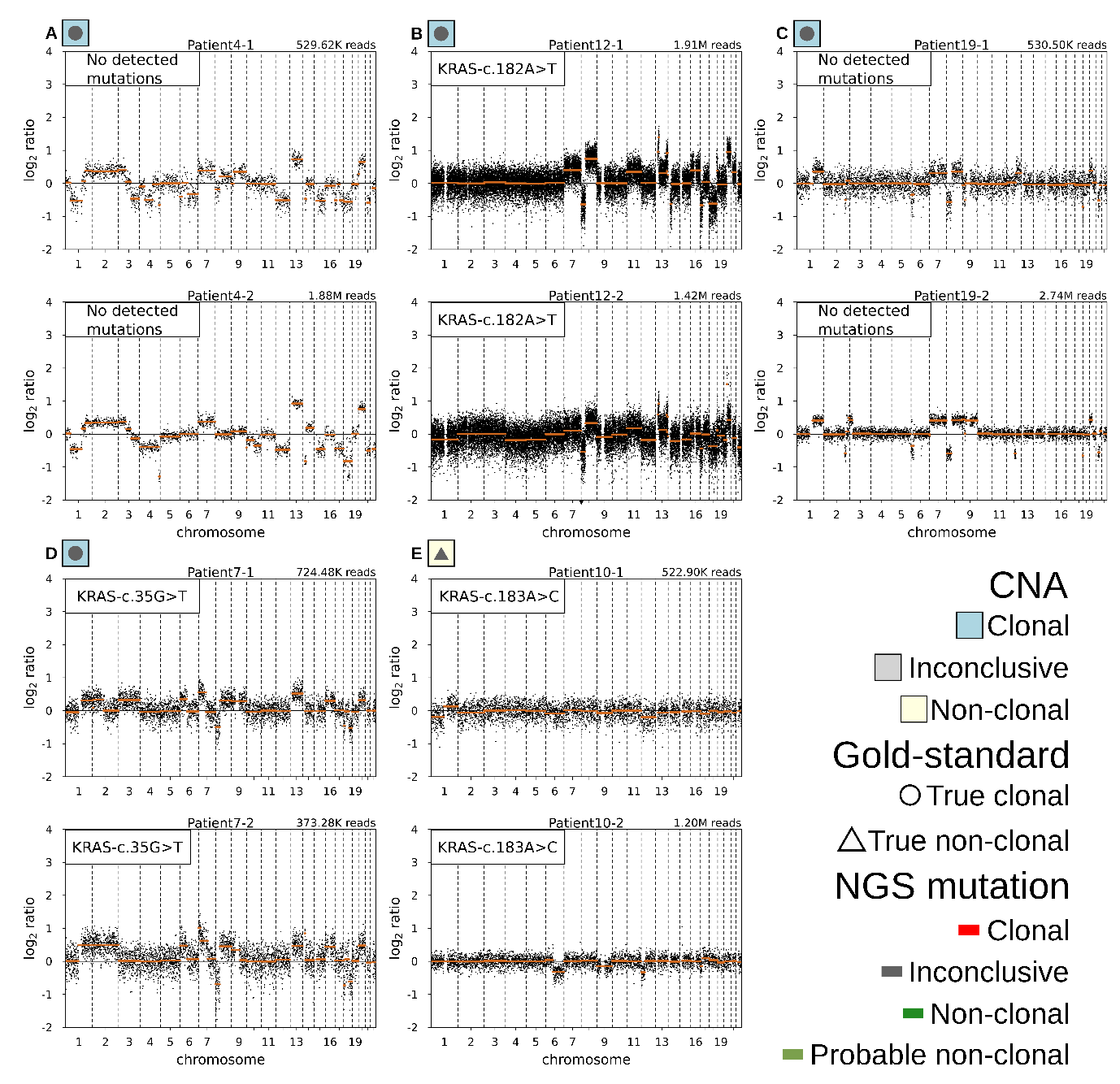
**

**Supplementary Figure 4. CNA profiles of AUMC diagnostic tumor pairs with inconclusive NGS mutation clonality or discordance with gold-standard clonality.** CNA profiles of AUMC diagnostic samples (A-E). CNA-, NGS mutation- and gold-standard clonality for each pair is indicated in the top left according to the color/shape legend in the bottom right. Black dots indicate log_2_ read counts (y-axis) and orange lines indicate segmentation values. Total binned read counts for each profile are indicated in the top right. The NGS mutation panel calls are indicated for each tumor in the top left.


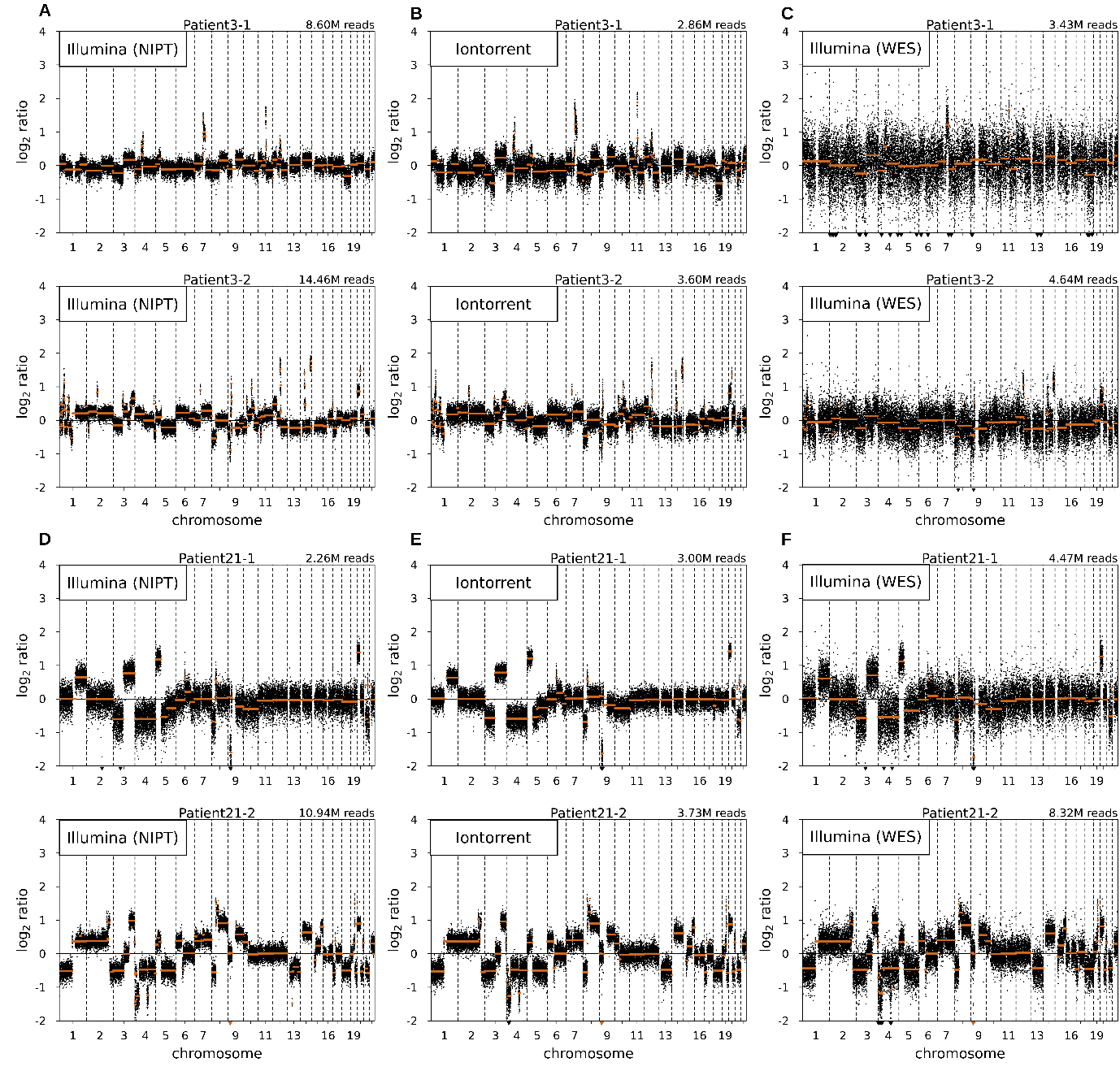


**Supplementary Figure 5. CNA profiles of AUMC tumor pairs using different sequencing platforms.** CNA profiles of two AUMC diagnostic sample pairs sequenced using the Illumina NIPT (A.D), Iontorrent (B,E) and Illumina WES (C,F) strategies. Black dots indicate log_2_ read counts (y-axis) and orange lines indicate segmentation values. Total binned read counts for each profile are indicated in the top right.

## **Supplementary Methods**

### Filtering and normalization of read counts derived from WES in the TRACERx cohort

For the TRACERx cohort, 100 kbp binned read counts were inferred from WES using CopywriteR (v2.20.0) (1). Subsequently, bins were corrected for GC bias and mappability bias and problematic bins were filtered from downstream analysis using the R package QDNAseq (v1.24.0) (2). In addition, CNAs that were called in more than 90% of all samples were discarded as artefacts. Further corrections were performed by dividing each bin with the mean copy number per bin in 32 TRACERx normal blood samples. Segmentation was performed using circular binary segmentations (CBS) implemented in the DNAcopy R-package (v1.74.1) (3). Copy number gains and losses were called using CGHcall (v2.62.0) (4).

### Bioinformatics and laboratory procedures for genome-wide CNAs for the in-house MC cohort

In the routine diagnostics of the Amsterdam UMC pathology department, under ISO 15189 accreditation, DNA samples were fragmented (Covaris™ ME220, Woburn, MA, USA) prior to barcoding. Library preparation was performed with slight adaptations over time. From 2016, the TruSeq DNA-kit v2 was used and sequencing performed using a 50 bp single-read modus on a HiSeq 4000 (Illumina San Diego, CA, USA) which includes 10 cycles of PCR amplification as described (2). In 2018 the shallow-WGS protocol changed to the PCR-free Ilumina Veriseq NIPT v2 workflow in combination with the NextSeq 500 (Illumina San Diego, CA, USA). Sequencing was performed with a target of 10 million reads and 2 samples run together with 94 samples in our NIPT diagnostic workflow (5). Shallow-WGS sequence reads were analyzed for genome-wide CNA by first aligning to the human reference genome build GRCh37/hg19 with BWA mem v0.7.10 (6). PCR duplicates (marked by Picard v1.111), as well as low-quality reads (MAPQ < 37), were filtered out using SAMtools v0.1.18 (7). CNA analysis was performed using the QDNAseq R package v1.24.0 (2) with a bin size of 100, 500 or 1000 kbp depending on the obtained sequence depth, which includes initial filtering (2). Segmentation was performed using CBS implemented in the DNAcopy R package v1.74.1 (3). Copy number gains and losses were called using CGHcall (v2.62.0) (4). CNAs called in more than 90% of all samples were also regarded as artefacts and filtered out.

### Bioinformatics and laboratory procedures for mutation calling in the in-house MC cohort

WES was performed for a selection of samples of the in-house MC cohort. Therefore, the KAPA HyperPlus Kit (KAPA Biosystems, Wilmington, MA) was used and sequenced on the NovaSeqX (Illumina), using 150 bp paired end sequencing with a target of 200x median exome coverage. The raw sequence reads were aligned and preprocessed using a previously published standard pipeline (8). For the six samples subjected to sWGS at both Ipatimub and Amsterdam UMC, genome-wide CNAs were also generated using CopywritR using the same procedure as described above but without normal corrections.

Mutation calling was performed using Mutect2 (v4.1.7.0) (9) and LoFreq (v2.1.3.1) (10). Only variants detected by both Mutect2 and LoFreq were selected. Variant calls with insufficient evidence were filtered based on the following criteria: minimum sequence depth (less than 15), minimum variant allele frequency (VAF) (less than 0.05), minimum variant supporting reads (less than 4), and minimal mapping quality (less than 20). Germline variants and platform-specific artifacts detected at least twice in our in-house reference WES dataset of 247 blood normal samples were filtered out. Germline variants detected at least once in either dbSNP (build 151) (11), gnomAD (v2.0.2) (12) or Hartwig Medical Foundation database of healthy individuals (v2.0) (13), were filtered out.

Since no matched-normal DNA was available for the in-house MC cohort, an excess of germline variants would remain in the dataset (Supplementary Figure 2A). To tackle this, we made use of the pairs where non-overlapping variants are per definition not germline variants, while the overlapping variants are a combination of both somatic and germline variants (Supplementary Figure 2A-C). For each patient, we first distinguished between overlapping and non-overlapping DNA variants of tumor pairs, followed by defining a probability distribution of somatic VAF values as a Gaussian distribution with parameters μ=mean(VAF_non-overlap_) and σ^2^=variance(VAF_non-overlap_) (Supplementary Figure 2D). To further differentiate somatic variants from germline variants among the overlapping variants, we calculated the probability of VAFs for each overlapping variant occurring in the aforementioned probability distribution (Supplementary Figure 2E) and selected only the top 50% of variants with the highest probabilities (Supplementary Figure 2F). Since VAFs are influenced by copy number gains or losses, only variants located in copy number neutral (i.e. diploid) genomic regions were used for the above analysis, as judged from the CNA profiles.

### Laboratory procedures for genome-wide CNAs in parallel with routine targeted panel NGS

We developed a workflow for shallow-WGS in parallel with routine targeted panel NGS using the IonTorrent workflow. First, 100 ng DNA of each WGS sample was fragmented at 37°C for 15 min using Ion Shear™ DNA fragmentation chemistry (ThermoFisher Scientific), followed by size selection with magnetic beads (AmpureXP, Agencourt). WGS libraries were subsequently prepared with the Ion Xpress Plus Fragment Library kit (ThermoFisher Scientific). WGS was performed with Ion GeneStudio sequencers. Thereby, WGS samples were combined with samples from routine targeted panel NGS on one 540 chip, totaling 8 samples.

### Classification of clonality using a conventional panel of mutations

From WES mutation data, calls overlapping with a conventional mutation panel were extracted. This panel covers 0.016Mb and is used for routine diagnostics at the Amsterdam UMC. The mutation calls obtained for each tumor pair were compared to classify clonality based on the adapted 2024 IASLC molecular classification algorithm (14, 15) (an overview of the algorithm is presented in Figure 1A). Thereto, tumor pairs with different oncogenic driver mion status were classified as ‘non-clonal’. Oncogenic driver mutations are defined as DNA variants in *EGFR*, *KRAS*, *BRAF* and *ERBB2* genes, which are designated as ‘likely oncogenic’ or ‘oncogenic’ in the OncoKB database (v4.20) (16). Tumor pairs with different *KRAS* driver mutation status were classified as ‘probable non-clonal‘, as proposed earlier (14). For tumor pairs with the same oncogenic driver status, other mutations (excluding oncogenic driver mutations) were compared. Tumor pairs with at least one shared mutation were classified as ‘clonal’. If no mutation was shared between tumor pairs, *TP53* mutation status was evaluated. If both tumors were *TP53* wildtype, the pair was classified as ‘inconclusive’ and if both tumors had a different *TP53* mutation status, the pair was classified as ‘probable non-clonal’.

## **Supplementary References**

1. Kuilman T, Velds A, Kemper K, Ranzani M, Bombardelli L, Hoogstraat M, et al. CopywriteR: DNA copy number detection from off-target sequence data. Genome Biol. 2015;16(1):49.

2. Scheinin I, Sie D, Bengtsson H, van de Wiel MA, Olshen AB, van Thuijl HF, et al. DNA copy number analysis of fresh and formalin-fixed specimens by shallow whole-genome sequencing with identification and exclusion of problematic regions in the genome assembly. Genome Res. 2014;24(12):2022-32.

3. Venkatraman ES, Olshen AB. A faster circular binary segmentation algorithm for the analysis of array CGH data. Bioinformatics. 2007;23(6):657-63.

4. van de Wiel MA, Kim KI, Vosse SJ, van Wieringen WN, Wilting SM, Ylstra B. CGHcall: calling aberrations for array CGH tumor profiles. Bioinformatics. 2007;23(7):892-4.

5. van Schendel RV, van El CG, Pajkrt E, Henneman L, Cornel MC. Implementing non-invasive prenatal testing for aneuploidy in a national healthcare system: global challenges and national solutions. BMC Health Serv Res. 2017;17(1):670.

6. Li H, Durbin R. Fast and accurate short read alignment with Burrows-Wheeler transform. Bioinformatics. 2009;25(14):1754-60.

7. Li H, Handsaker B, Wysoker A, Fennell T, Ruan J, Homer N, et al. The Sequence Alignment/Map format and SAMtools. Bioinformatics. 2009;25(16):2078-9.

8. Los-de Vries GT, Stevens WBC, van Dijk E, Langois-Jacques C, Clear AJ, Stathi P, et al. Genomic and microenvironmental landscape of stage I follicular lymphoma, compared with stage III/IV. Blood Adv. 2022;6(18):5482-93.

9. Cibulskis K, Lawrence MS, Carter SL, Sivachenko A, Jaffe D, Sougnez C, et al. Sensitive detection of somatic point mutations in impure and heterogeneous cancer samples. Nat Biotechnol. 2013;31(3):213-9.

10. Wilm A, Aw PP, Bertrand D, Yeo GH, Ong SH, Wong CH, et al. LoFreq: a sequence-quality aware, ultra-sensitive variant caller for uncovering cell-population heterogeneity from high-throughput sequencing datasets. Nucleic Acids Res. 2012;40(22):11189-201.

11. Sherry ST, Ward MH, Kholodov M, Baker J, Phan L, Smigielski EM, et al. dbSNP: the NCBI database of genetic variation. Nucleic Acids Res. 2001;29(1):308-

12. Karczewski KJ, Francioli LC, Tiao G, Cummings BB, Alfoldi J, Wang Q, et al. The mutational constraint spectrum quantified from variation in 141,456 humans. Nature. 2020;581(7809):434-43.

13. Martinez-Jimenez F, Movasati A, Brunner SR, Nguyen L, Priestley P, Cuppen E, et al. Pan-cancer whole-genome comparison of primary and metastatic solid tumours. Nature. 2023;618(7964):333-41.

14. Janssen J, Andrade Barbosa B, Machado JC, Hofman P, Kim Y, Ylstra B, et al. Performance and considerations in the use of diagnostic mutation panels for clonality testing in non-small-cell lung carcinoma. ESMO Open. 2025;10(5):105072.

15. Chou TY, Dacic S, Wistuba I, Beasley MB, Berezowska S, Chang YC, et al. Differentiating Separate Primary Lung Adenocarcinomas From Intrapulmonary Metastases With Emphasis on Pathological and Molecular Considerations: Recommendations From the International Association for the Study of Lung Cancer Pathology Committee. J Thorac Oncol. 2024;20(3):311-30.

16. Chakravarty D, Gao J, Phillips SM, Kundra R, Zhang H, Wang J, et al. OncoKB: A Precision Oncology Knowledge Base. JCO Precis Oncol. 2017;2017.
